# KINLESSNESS, RACE, AND QUALITY OF CARE AT THE END OF LIFE: FINDINGS FROM NATIONAL HEALTH AND AGING TRENDS STUDY

**DOI:** 10.1093/geroni/igad104.3446

**Published:** 2023-12-21

**Authors:** Yaolin Pei, Xiang Qi, Zheng Zhu, Bei Wu

**Affiliations:** New York University, New York City, New York, United States; New York University, New York City, New York, United States; New York University, New York City, New York, United States; New York University, New York City, New York, United States

## Abstract

Close family members provided the majority of end-of-life (EOL) caregiving. The number of older adults who are kinless (without close family/partnerships) are increasing. We therefore examined the association between kinlessness and quality of EOL care among older adults, and the moderating role of race in this association. Data were derived from the combined Round 2 to Round 11 of the National Health and Aging Trends Study. 3066 older adults who aged 65 and above and were living in the community or residential care at last interview were included in the working sample. Kinlessness was defined as lacking children and a spouse/partner. The primary outcome was quality of care at the end of life. Logistic regression models ware used to examine the association between kinlessness, race, and quality of care at the end of life. A total of 8.0% of decedents were kinless at the end of life. Kinless older adults were less likely to receive excellent EOL care than those with kin. Having at least one child or spouse/partner weakened race disparities in EOL care quality among older adults. These results point to a significant disadvantage for the minority kinless older adults, who had worse EOL care quality. Practice interventions may include a culture match between older adults and providers. Form a policy standpoint, guardianship is needed to enable kinless older adults to receive high-quality EOL care.

